# A Controllable Aptamer-Based Self-Assembled DNA Dendrimer for High Affinity Targeting, Bioimaging and Drug Delivery

**DOI:** 10.1038/srep10099

**Published:** 2015-05-11

**Authors:** Huimin Zhang, Yanli Ma, Yi Xie, Yuan An, Yishun Huang, Zhi Zhu, Chaoyong James Yang

**Affiliations:** 1 The MOE Key Laboratory of Spectrochemical Analysis & Instrumentation, Collaborative Innovation Center of Chemistry for Energy Materials, State Key Laboratory of Physical Chemistry of Solid Surfaces, the Key Laboratory for Chemical Biology of Fujian Province, Department of Chemical Biology, College of Chemistry and Chemical Engineering, Xiamen University, Xiamen 361005, P. R. China

## Abstract

Targeted drug delivery is important in cancer therapy to decrease the systemic toxicity resulting from nonspecific drug distribution and to enhance drug delivery efficiency. We have developed an aptamer-based DNA dendritic nanostructure as a multifunctional vehicle for targeted cancer cell imaging and drug delivery. The multifunctional DNA dendrimer is constructed from functional Y-shaped building blocks with predesigned base-pairing hybridization including fluorophores, targeting DNA aptamers and intercalated anticancer drugs. With controllable step-by-step self-assembly, the programmable DNA dendrimer has several appealing features, including facile modular design, excellent biostability and biocompatibility, high selectivity, strong binding affinity, good cell internalization efficiency, and high drug loading capacity. Due to the unique structural features of DNA dendrimers, multiple copies of aptamers can be incorporated into each dendrimer, generating a multivalent aptamer-tethered nanostructure with enhanced binding affinity. A model chemotherapeutic anticancer drug, doxorubicin, was delivered via these aptamer-based DNA dendrimers and exerted a potent toxicity for target cancer cells (human T cell acute lymphoblastic leukemia cell line) with low side effects for the non-target cells (human Burkitt’s lymphoma cell line). This controllable aptamer-based DNA dendrimer is a promising candidate for biomedical applications.

Chemotherapy is widely used for cancer treatment using traditional small molecule drugs. However, the chemotherapeutic drugs may lead to serious toxic side effects and inefficient delivery to tumor tissues due to poor water solubility, nonspecific distribution and systemic toxicity^1^,^2^. Thus, development of desirable therapeutics which can penetrate biological barriers, distinguish normal and diseased tissues, and intelligently respond to the tumor microenvironment for on-demand drug release, is an urgent need^3^. Nanotechnology applied in medicine, known as nanomedicine, has become a promising approach for efficient cancer therapeutics. This technology uses precisely engineered materials at the scale of 1–100 nm to develop novel therapeutic and diagnostic modalities^4^,^5^. For successful nanomedicine, nanoparticle sizes and surface properties must be controlled, and targeting ligands must be incorporated for site-specific on-demand release of pharmacologically active agents at therapeutically optimal rates and dose regimens^6^.

Various types of nanoparticles, including self-assembled polymers and metal nanoparticles, have been used as potential diagnostic and therapeutic agents, representing a promising breakthrough^7^,^8^,^9^. However, most inorganic and organic nanomaterials suffer from multiple drawbacks, such as limited biocompatibility and inability to engineer spatially addressable surfaces that can be utilized for multifunctional activities. Alternatively, DNA-based nanostructures are promising materials for biomedical applications^10^, because of their excellent biocompatibility, specific base pairing interactions, automated synthesis, and programmability^11^. For example, uniform sized DNA tetrahedrons equipped with immune-stimulatory CpG oligonucleotides or small interface RNA have shown enhanced intracellular immunoregulation or gene delivery with excellent biostability and biocompatibility^12^,^13^ Likewise, triangular DNA origami loaded with the anticancer drug doxorubicin exhibited improved anti-tumor efficacy and lower systemic toxicity *in vivo* compared to anti-cancer drugs^14^.

Among the diverse DNA nanostructures, DNA dendrimers have attracted increasing interest in the past decade due to their monodispersity, excellent stability, globular shape, and highly branched and porous structures^15^. Mintzer and coworkers used DNA dendrimers for delivery of functional molecules, such as the CpG motif^16^, into cells with excellent intracellular uptake via passive delivery. Tan and coworkers used Y-shaped monomers and DNA linkers to form DNA hydrogel for targeted gene therapy^17^. Although passive delivery is useful for cancers with leaky vasculatures, it is not suitable for other types of cancers, such as leukemia, that require specific targeting. In this regard, incorporation of a ligand which targets a particular cell receptor to facilitate receptor-mediated endocytosis could provide enhanced versatility for the treatment of a variety of diseases^18^.

Aptamers are single-stranded DNA or RNA oligonucleotides screened by a process called Systematic Evolution of Ligands by Exponential Enrichment (SELEX)^19^. Aptamers have excellent advantages as targeting ligands, such as high target affinity, excellent specificity and low immunogenicity. Aptamers can recognize a large range of targeting molecules, including organic and inorganic small molecules, proteins, cells and even tissues. Furthermore, the easy synthesis and functionalization of aptamers make it possible to design various aptamer chimeras, such as aptamer-dye, aptamer-drug, aptamer-biomolecule and aptamer-nanomaterial conjugates, to generate diversified molecular probes in sensing, imaging and targeted therapy^20^,^21^. Most importantly, aptamers can be easily designed and integrated into 3D nucleic acid structures without any need of chemical modification. Combined with the dendritic DNA structures and designed hybridization, it is possible to embed a variety of ligands and functional reagents to generate multifunctional nano-platforms.

In this work, we designed an aptamer-based DNA dendrimer as a multifunctional nanostructure for biomedical applications. In our proof-of-principle study, we have successfully incorporated functional domains, including aptamers, fluorophores, and drug loading sites, into DNA dendrimers to achieve selective cancer cell recognition, bioimaging, and targeted anticancer drug delivery. The aptamer used in this study, sgc8, which selectively recognizes cell membrane protein PTK7^22^, was artificially designed to spontaneously hybridize with building blocks of the outer shell of the DNA dendrimer. PTK7-overexpressed cell lines, human T cell acute lymphoblastic leukemia cell line (CCRF-CEM) and cervical cancer HeLa cell line, were chosen as targets, and human Burkitt’s lymphoma cell line (Ramos) with low expression of PTK7 was used as a control. By combining the advantages of DNA dendrimers and the sgc8 aptamer, these nanostructures can selectively distinguish and be internalized by the target cells. Because of their abundant double-stranded sequences, DNA dendrimers have high capacity to load intercalating therapeutic drugs. In this work, doxorubicin (Dox), a model chemotherapeutic anticancer drug, was loaded into DNA dendrimer for evaluating the targeted therapeutic effect. This aptamer-based self-assembled DNA dendrimer provides the advantages of facile modular design and assembly, high programmability and biocompatibility, as well as selective recognition. With controllable functional groups, these DNA dendrimers have remarkable potential for application in multifunctional bioimaging and drug delivery.

## Results and Discussions

### Design, preparation and characterization of multifunctional DNA Dendrimer

**Figure 1:**
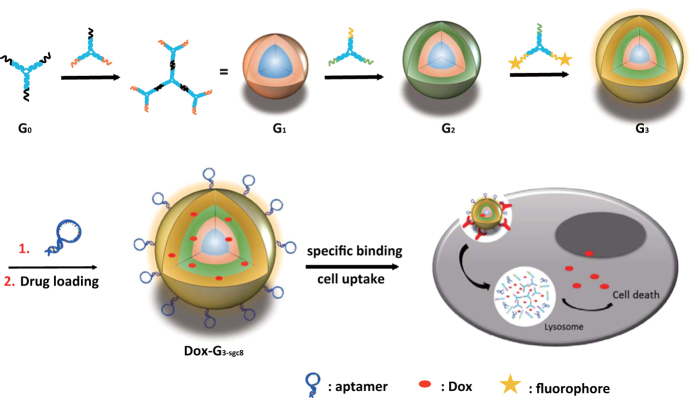
Working principle of aptamer-based dendritic DNA nanostructure. The final generation Dox-G_3-sgc8_ is designed with various functional groups, including fluorophores, targeting ligands and anticancer drugs to endow the DNA dendrimer with the capability of cancer cell recognition, imaging and drug delivery.

**Figure 2:**
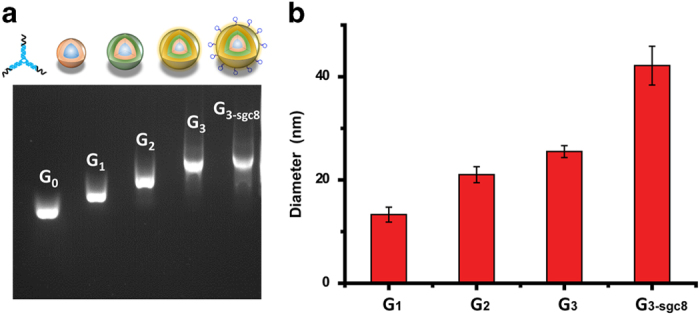
(**a**) Agarose gel electrophoresis of different generation of DNA dendrimers G_0_ is Y-DNA (Y_0_); G_1_–G_3-sgc8_ are DNA dendrimers. (**b**) Diameter of G_1_, G2, G3, G_3-sgc8_ dendrimers measured by DLS analysis.

Multifunctional DNA dendrimers were prepared from three-armed Y-shaped DNA monomers using an enzyme-free, step-by-step base-pairing assembly strategy^23^. Y-shaped DNA monomers contained 13-base sticky-end segments, which further hybridized with other Y-DNAs as each generation was added. The Y-DNA called Y_0_ was assembled from the hybridization of three single strands, Y_0a_, Y_0b_, and Y_0c_, by slowly cooling from 95 ^o^C to 4 ^o^C in 91 minutes. The others, Y_1_, Y_2_, and Y_3,_ were prepared according to the same procedure from their three respective single-stranded sequences. The as prepared Y-DNAs were then characterized by native-PAGE gel electrophoresis (Fig. S1) and ready to use without purification. Different generations of DNA dendrimer (G_n_) were prepared from Y-shaped building blocks by layer-by-layer assembly. The first generation G_0_ was the initial building block Y_0_. For G_n_, it was synthesized by mixing G_n-1_ and Y_n_ with the ratio of 1: 3 × 2^n−1^ (n > 1) at room temperature for 1 hour. Based on the G_3_ structure, aptamers (sgc8) with sticky-end pairing with Y_3_ were added in the solution to form the aptamer-based DNA self-assembled nanostructure G_3-sgc8_, as shown in Fig. 1. Each generation was characterized by agarose gel electrophoresis and dynamic light scattering (Fig. 2) and directly used without purification. Only one major band for each generation was detected by gel electrophoresis suggesting the formation of highly pure DNA dendrimer. The band mobility decreased with increasing generation, suggesting the success of self-assembly (Fig. 2a). DLS measurement showed the average diameter of G_1_, G_2_, G_3_ and G_3-sgc8_ to be 13.7, 21.0, 24.3 and 43.8 nm, respectively (Fig. 2b). These data verified that higher generations of DNA dendrimer were heavier in mass and larger in size. To confirm the structure of DNA dendrimer, atomic force microscopy (AFM) was also used to characterize G_3-sgc8_. The measured diameter was correlated to the result of dynamic light scattering measurement. And it showed G_3-sgc8_ as a spherical structure which indicated the formative assembly was a DNA dendrimer (Fig. S2).

### Selective Recognition of Target Cancer Cells by FITC labeled G_3-sgc8_

**Figure 3:**
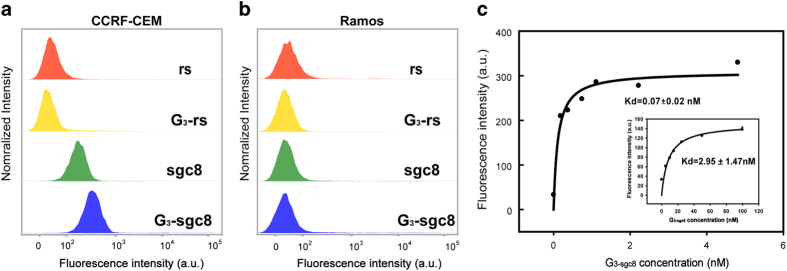
Selective cancer cell recognition by multifunctional dendrimer. Flow cytometry results demonstrated the selective recognition of G_3-sgc8_ (FITC-incorporated on G_3_) to target CCRF-CEM cells (**a**) but not to control Ramos cells (**b**). (**c**) Binding affinity of G_3-sgc8_ and fluorescein-labeled free sgc8 (inside figure) to CCRF-CEM cells. G_3-sgc8_ showed about 40-fold higher binding affinity than single aptamer.

After confirming the successful formation of DNA dendrimers, the cancer cell recognition property of G_3-sgc8_ was investigated. With aptamer sgc8 coating on the surface, G_3-sgc8_ is expected to specifically recognize target cancer cell line CEM. In this study, an organic dye, FITC, was labeled on Y_3_ to stain G_3-sgc8_ with green fluorescence to investigate specific binding. Fig. 3a,b shows the flow cytometric comparison of target (CCRF-CEM) cells and control negative (Ramos) cells after incubation with random DNA library (lib), individual aptamer (sgc8), dendrimer decorated with random sequences (G_3-rs_) and aptamer-embedded dendrimer (G_3-sgc8_). Weak fluorescence was observed with random DNA and random DNA dendrimer for both CEM and Ramos cells, indicating low non-specific binding. There was a noticeable change in the fluorescence signal observed for CEM cells treated with free sgc8 because of the specific binding between aptamer and target cell line, while CEM cells treated with G_3-sgc8_ showed significantly higher fluorescence intensity than aptamer stained cells. No significant change in fluorescence intensity was observed for Ramos cells with either free sgc8 or G_3-sgc8_, further confirming the specific recognition of G_3-sgc8_ to target CEM cells. The binding affinities of free sgc8 and G_3-sgc8_ were determined by incubating CCRF-CEM cells with varying concentration of aptamer probes on ice for 30 min. As demonstrated in Fig. 3c, G_3-sgc8_ showed a significantly enhanced binding affinity (*K*_d_ = 0.07 ± 0.02 nM) compared to individual sgc8 aptamer (*K*_d_ = 2.95 ± 1.47 nM). G_3-sgc8_ showed at least a 40-fold enhancement in fluorescence intensity compared to the free aptamer with CCRF-CEM cells. This high binding affinity of G_3-sgc8_ is indicative of multivalent-mediated enhancement of binding affinity, because multiple aptamers on the outer-shell dendrimer can each recognize the receptors on the cell surface.

**Figure 4:**
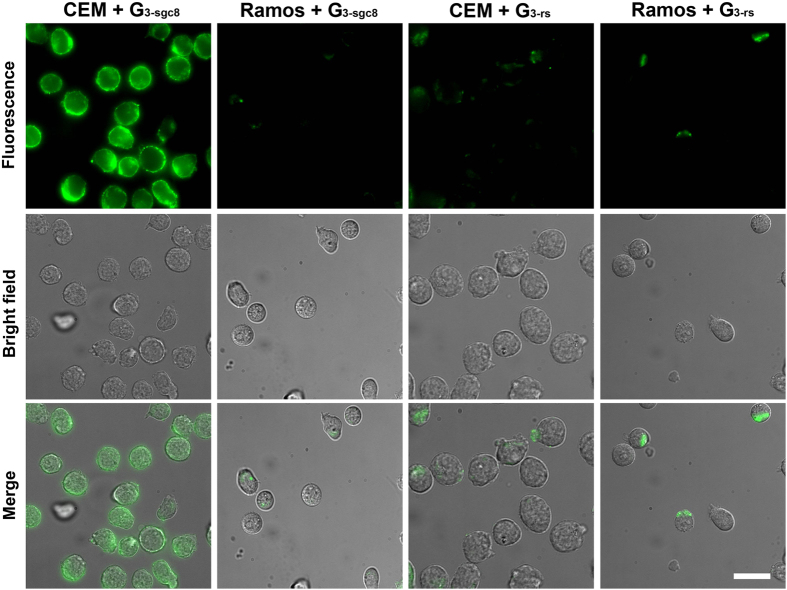
Microscopic images of target cell CEM and control cell Ramos incubated with G_3-sgc8_ and G_3-rs_. Green fluorescent G_3-sgc8_ was accumulated in CEM but not in Ramos. Scar bar: 25 μm.

Bioimaging is a visual method to investigate specific recognition and cellular trafficking of DNA assembled-nanoparticles. A strong green fluorescence signal was observed by the DeltaVision Elite cell imaging system after incubating G_3-sgc8_ with CEM at 37 °C for 2 h (Fig. 4). In contrast, a weak fluorescence signal was observed for G_3-rs_ and Ramos cells. Previous work suggested that sgc8 aptamer enters the targeted cell line, such as CEM and HeLa, through receptor PTK7-mediated endocytosis. A colocalization assay was performed in this work to track the final destination of G_3-sgc8_ in live HeLa cells. Most of the green fluorescence signal from G_3-sgc8_ overlapped with the red fluorescence signal generated from a lysotracker (a lysosome marker, Fig. S3), indicating that aptamer-based DNA dendrimer can recognize and internalize into target cancer cells through receptor-mediated endocytosis instead of passive delivery, an important property for use as a multifunctional nano-platform for efficient delivery of imaging and therapeutic reagents into the cytoplasm.

### Selective cytotoxicity of anticancer drug-loaded G_3-sgc8_

Benefitting from the large number of hybridized DNA base pairs, the DNA dendrimer is spatially well equipped for loading chemical anticancer drugs such as doxorubicin (Dox). Dox can preferentially insert between G-C pairs, resulting in the quenching of Dox fluorescence due to Förster resonance energy transfer. We then determined Dox intercalation by monitoring Dox fluorescence intensity changes to evaluate the amount of Dox loaded into the G_3-sgc8_. Dox fluorescence was dramatically quenched by G_3-sgc8_ with a molar ratio of 666/1, indicating a high loading capacity of 10 nM G_3-sgc8_ with ∼6.66 μM Dox, as shown in Fig. S6. We next investigated the release kinetics of Dox loaded into G_3-sgc8_ by detecting the intensity of Dox fluorescence. The G_3-sgc8_ (2 nM) with a drug payload of 666 nM released less than 7% of the DOX after 60 hours in a physiological environment (pH = 7.4 PBS buffer, Fig. S7). Therefore, the Dox payload was sufficiently stable to prevent drug leakage during the circulation in blood.

**Figure 5:**
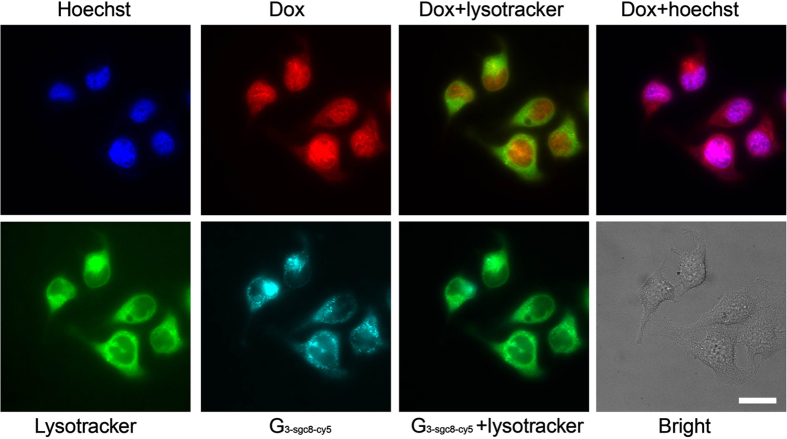
Subcellular distribution of Dox (red) loaded G_3-sgc8-cy5_ (cyan). Hoechest (blue) and Lysotracker Green (green) were used to stain the cell nuclei and acidic organelles. Cells were imaged using a 60x oil-immersion objective. The merged images were used to confirm that drug had released and escaped into the nucleus within 2.5 hours, but the nanostructure still stayed in the lysosome. Scar bar: 20 μm.

To investigate the drug transport into cell and drug release, the uptake and distribution of Dox-G_3-sgc8-cy5_ were studied with target HeLa cells using microscope imaging. We incubated Dox-G_3-sgc8-cy5_ with HeLa cells for 30 min (Fig. S4) and 2.5 hours (Fig. 5), then washed 3 times with PBS buffer, followed by staining with Hoechst and lysotracker. As shown in Fig. 5, G_3-sgc8-cy5_ was still predominantly localized in lysotracker-labeled acidic organelles after 2.5 hours, while most of the inserted Dox had escaped from the dendrimer and appeared colocalized with Hoechst in the nucleus. We believe that the transported Dox-G_3-sgc8-cy5_ first entered cells via receptor-mediated endocytosis, and then escaped from the endosome, finally localizing in the lysosome. The acidic lysosome environment and enzyme catalysis facilitated the rapid release of the loaded anticancer drug, which traveled to the nucleus.

**Figure 6:**
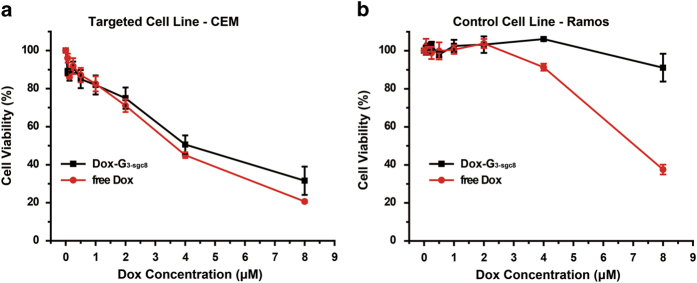
MTS assay results showing selective cytotoxicity of Dox delivered by DNA dendrimer in target CEM cells (**a**) but much less in nontarget Ramos cells (**b**), in contrast to nonselective cytotoxicity of free Dox in both target cells and nontarget cells. The selective cytotoxicity of Dox delivered by DNA dendrimer indicates the capability of DNA dendrimers for targeted drug delivery.

The *in vitro* cytotoxicity of Dox-G_3-sgc8_ and free Dox were evaluated by the 3-(4, 5-dimethylthiazol-2-yl)-5-(3-carboxymethoxyphenyl)-2-(4-sulfophenyl)-2H-tetrazolium (MTS) assay. In target CEM cells, Dox-G_3-sgc8_ showed the similar inhibition of cell proliferation as free Dox (Fig. 6a), while in non-target cell Ramos, Dox-G_3-sgc8_ showed significantly less inhibition of cell proliferation than free Dox. In contrast, for free drug, there was no drug selectivity in either target or non-target cancer cells. These results indicate that aptamer-induced targeted internalization enhanced site-specific drug delivery and suggest that our aptamer-based DNA dendrimer is an excellent vehicle for targeted cancer therapy.

## Conclusions

We have demonstrated an aptamer-based DNA dendrimer as an anticancer drug carrier specific recognition behavior and selective cytotoxicity to target cancer cell lines. This designable DNA nanostructure can be equipped with different functional groups using oligonucleotide base pairing without complicated chemical modification. For biomedical applications, the DNA dendrimer has several remarkable features: (1) *Facile design and preparation*. All the DNA assembly mentioned in this paper occurs at room temperature by mixing different building blocks in a fixed ratio, and the size can be controlled by adding more or fewer generations. (2) *Multifunctionality*. Different functional domains, including imaging dyes, targeting ligands and inserted anticancer drugs, are integrated in a single platform to fulfill diverse demands. (3) *Good biocompatibility*. Our DNA dendrimer shows very little toxicity without drug cargo (Fig. S7), while it is very toxic to target cells when carrying anticancer drugs. (4) *Excellent stability*. The stability of dendritic DNA nanostructure has been examined in many previous publications. For example, there was no structural change after direct treatment with endonuclease DNase I (1 U/mL, a considerably higher concentration than would be found in living cells)^24^. The DNA dendrimer also has excellent and stable loading capacity for anticancer drugs, as shown by a 60-hour test in physiology environment (Fig. S6). With these remarkable features, together with the attractive properties of cancer cell specific recognition, imaging and drug delivery, the multifunctional DNA dendrimer offers a promising new modality for selective multimodal cancer theranostics.

## Methods

### Materials

All oligonucleotides were purchased from Sangon (Shanghai, China) and used without further purification. Lysotracker and Hoechst were purchased from Life Technology (Beijing, China). RPMI-1640 and DMEM Medium were obtained from HyClone (Beijing, China). Fetal bovine serum (FBS) was obtained from Gibco through Life Technology (Beijing, China). Doxorubicin was purchased from Huafeng United Technology Co., Ltd. (Beijing, China). CellTiter 96^®^ AQueous Non-Radioactive Cell Proliferation Assay (MTS) was purchased from Promega, Madison, WI, USA.

### Cell culture

CCRF-CEM (CCL-119, T-cell line, human acute lymphoblastic leukemia), HeLa (Human cervical cancer cell line) and Ramos (CRL-1596, B-cell line, human Burkitt’s lymphoma) cells were obtained from American Type Culture Collection. CCRF-CEM and Ramos were cultured in RPMI 1640 and HeLa was cultured in DMEM, which contained 10% fetal bovine serum (FBS, 10%), and penicillin–streptomycin (100 IU/mL) at 37 ^o^C in a humid atmosphere with 5% CO_2_.

### Synthesis of DNA dendrimer

Y-shaped DNA was assembled according to the method as reported^23^. For preparation of Y-shaped DNA (e.g. Y_0_), three strands (Y_0a_, Y_0b_, Y_0c_) were mixed in the phosphate buffer (50 mM phosphate, 100 mM Na^+^, pH = 8.0) and the final concentration of each strand was 20 uM. Then, the mixture was heat to 95 ^o^C for 2 min and cooled to 4 ^o^C at a rate of 1 ^o^C/min. To prepare DNA dendrimers (G_n_), Y_0_ (G_0_) and Y_1_ were mixed at a 1:3 molar ratio and the mixture was kept at room temperature for 2 h and at 4 ^o^C for 2 h to prepare G_1_. G_n_ was prepared using the same method by mixing G_n-1_ and Y_n_ in a ratio of 1:3^2n−1^.

### Characterization of DNA dendrimer

The Y-shaped DNAs used in the experiment were characterized by 10% native PAGE at 75 V in 1 × TBE buffer for 2 h on ice. They were all directly used for further assembly without purification. G_n_ was characterized by 1% agarose gel at 55 V in 1 × TAE buffer for 60 min on ice. In DLS experiments, described specifically for G_n_, 2 μL solution of G_n_ (50 mM, pH 8.0 phosphate buffer with 100 mM NaCl) was diluted to 200 μL by the same phosphate buffer and characterized by DLS to give the radius of G_n_. AFM imaging was performed on an Agilent 5500ILM SPM (Agilent Technologies, Inc.) equipped with a N9520A scanner with a scan size of 10 μm in x−y and 2.08 μm in z directions. Gold coated silicon probes (NT-MDT) with a nominal force constant of 0.01−0.08 N/m (CSG/11 Au) and 5.5−22.5 N/m (NSG/10) were used for force measurements and imaging, respectively. In a typical experiment, 2 μL sample solution was dropped onto the cleaved mica, left standing for 1 min, and then removed by aspiration. Then 10 μL water was dropped onto the surface and removed using a stream of nitrogen.

### Flow Cytometric Analysis

For the fluorescence analysis, Y_3a_ and Y_3b_ were labelled with FITC to generate a fluorescent dendrimer. To demonstrate the targeting capabilities of aptamer-conjugated dendrimer toward specific cells, fluorescence measurements were performed using a flow cytometer (BD Biosciences FACSVerse^TM^ cytometer), according to the following procedure: Approximately 1 × 10^5^ cells of each type were suspended with 200 μL BB buffer (1 × PBS, 0.55 mM MgCl_2_, pH = 7.4) in individual test tubes. To the cell samples, 2 μL of the G_3-aptamer_ solution (C_aptamer_ = 200 nM) was added, and the mixture was incubated at room temperature for 30 min. After incubation, the cells were washed twice by centrifugation with 0.5 mL buffer and resuspended in 0.2 mL buffer. The fluorescence was determined by counting 10,000 events. The fluorescein-labeled G_3-rs_ was used as a negative control.

The binding affinities of free and dendrimer-conjugated aptamer probes were determined by incubating CCRF-CEM cells (1 × 10^5^) at room temperature for 30 min in the dark with varying concentrations of aptamer probes in a 200 μL volume of buffer. Cells were then washed twice with 0.5 mL buffer, suspended in 0.2 mL buffer, and subjected to flow cytometric analysis within 30 min. All of the experiments for the binding assay were repeated two times. The equilibrium dissociation constants (K_d_) of the aptamer-cell interaction were obtained by fitting the dependence of fluorescence intensity of specific binding on the concentration of the aptamers to the equation Y = B_max_X/ (K_d_ + X), using SigmaPlot (Jandel, San Rafael, CA).

### Cell Imaging

For cell imaging, the treatment steps for cell incubation were the same as described in *Flow Cytometric Analysis*. Ten microliters of cell suspension bound with G_3-rs_ or G_3_-_sgc8_ was dropped on a thin glass slide placed above an objective on the confocal microscope. HeLa cell line was cultured in the confocal culture plate. G_3-sgc8_ was directly added to FBS-free medium and incubated for 2 h. Then washed twice with buffer and 200 μL PBS buffer was added to keep cells alive. For colocalization with lysosome, Lysotracker with a standard concentration was added and incubating in 37 ^o^C for 30 min. The Lysotracker was removed by washing and ready-to-use Hoechst solution was added (1 ug/mL in PBS buffer), followed by incubation at room temperature for 5 min. A 60x oil-immersion objective on a Nikon inverted microscope linked to a DeltaVision^TM^ deconvolution-imaging system (Applied Precision, Seattle, Washington) and a Leica TCS SP5 microscope with 100x oil-immersion objective (Leica Microsystems CMS GmbH, Germany) were used for imaging.

### Drug loading into G_3-aptamer_

To load Dox into *G*_*3-aptamer*_, Dox and *G*_*3-aptamer*_ were mixed with special ratio and stayed at room temperature to allow saturation of drug loading. To evaluate the amount of Dox (doxorubicin) loaded into the G_3-aptamer_, we measured Dox intercalation by monitoring Dox fluorescence intensity changes. When a fixed concentration of Dox was incubated with an increasing molar ratio of the G_3-aptamer_, a sequential decrease was found in the fluorescence intensity of Dox (Fig. S2), due to Förster resonance energy transfer between Dox molecules when intercalated into the DNA duplex. According to Fig. S2, 1 μL G_3-aptamer_ can tolerate 0.5 nmol Dox. To prevent leakage, we chose a Dox/G_3-aptamer_ of 333 as a better concentration for drug loading.

### Cytotoxicity test

The cytotoxicities of G_3-aptamer_, free drug, or drug-G_3-aptamer_ complexes were evaluated using CellTiter 96^®^ AQueous Non-Radioactive Cell Proliferation Assay (MTS). Cells (1 × 10^4^ CEM or Ramos cells/well) were treated with G_3-aptamer_, free drug, or drug-G_3-aptamer_ complexes in FBS-free medium. After incubation for 2 h in a cell culture incubator, supernatant medium was removed, and fresh medium (10% FBS, 100 μL) was added for further cell growth (24 h). Then MTS reagent (20 μL) was added to each well and incubated for 1–2 h at 37 ^o^C. The absorbance (490 nm) was recorded using a model 680 BioRad plate-reader (Bio-Rad, Hertfordshire, UK).

## Author Contributions

H.Z. designed, performed research and wrote the mauscript, Y.M., Y.X. prepared the Fig. 1–3, Y. A., Y. H. prepared the Fig. 4–5 and the supporting information, Z.Z. and C.J.Y. conceived the project, supervised the project and revised the manuscript.

## Additional Information

**How to cite this article**: Zhang, H. *et al*. A Controllable Aptamer-Based Self-Assembled DNA Dendrimer for High Affinity Targeting, Bioimaging and Drug Delivery. *Sci. Rep.* **5**, 10099; doi: 10.1038/srep10099 (2015).

## Supplementary Material

Supplementary Information
