# Supplementary material for: A Controllable Aptamer-Based Self-Assembled DNA Dendrimer for High Affinity Targeting, Bioimaging and Drug Delivery
Source: Sci Rep. 2015 May 11;5:10099. doi: 10.1038/srep10099 (PMC4431499; doi:10.1038/srep10099)
Supplement: Supplementary Information [file srep10099-s1.pdf]

Supporting Information

A Controllable Aptamer-Based Self-Assembled DNA Dendrimer for High Affinity Targeting, Bioimaging and Drug Delivery

*Huimin Zhang, Yanli Ma, Yi Xie, Yuan An, Yishun Huang, Zhi Zhu and Chaoyong James Yang**

The MOE Key Laboratory of Spectrochemical Analysis & Instrumentation, State Key Laboratory of Physical Chemistry of Solid Surfaces, the Key Laboratory for Chemical Biology of Fujian Province, Department of Chemical Biology, College of Chemistry and Chemical Engineering, Xiamen University, Xiamen 361005, P. R. China.

E-mail: cyyang@xmu.edu.cn

Table S1 Sequences of Oligonucleotides Used in This Work

Oligonucleotide	Sequence (from 5' to 3')
Y_{0a}	GAC CGA TGG ATG ACC TGT CTG CCT AAT GTG CGT CGT AAG
Y_{0b}	GAC CGA TGG ATG ACT TAC GAC GCA CAA GGA GAT CAT GAG
Y_{0c}	GAC CGA TGG ATG ACT CAT GAT CTC CTT TAG GCA GAC AGG
Y_{1a}	GAA GCC ACT CTG ACC TGT CTG CCT AAT GTG CGT CGT AAG
Y_{1b}	GAA GCC ACT CTG ACT TAC GAC GCA CAA GGA GAT CAT GAG
Y_{1c}	TCA TCC ATC GGT CCT CAT GAT CTC CTT TAG GCA GAC AGG
Y_{2a}	GAC ACA CTG AGG TCC TGT CTG CCT AAT GTG CGT CGT AAG
Y_{2b}	GAC ACA CTG AGG TCT TAC GAC GCA CAA GGA GAT CAT GAG

Y_{2c}	TCA GAG TGG CTT CCT CAT GAT CTC CTT TAG GCA GAC AGG
Y_{3a}	TAC TCG AGA CAT A CC TGT CTG CCT AAT GTG CGT CGT AAG
Y_{3b}	TAC TCG AGA CAT A CT TAC GAC GCA CAA GGA GAT CAT GAG
Y_{3c}	ACC TCA GTG TGT C CT CAT GAT CTC CTT TAG GCA GAC AGG
Sgc8-linker	TAT GTC TCG AGT ATT TTT ATC TAA CTG CTG CGC CGC CGG GAA AAT ACT GTA CGG TTA GA
RS-linker	TAT GTC TCG AGT ATT TTT NNN NNN NNN NNN NNN NNN NNN NNN NNN NNN NNN NNN NN

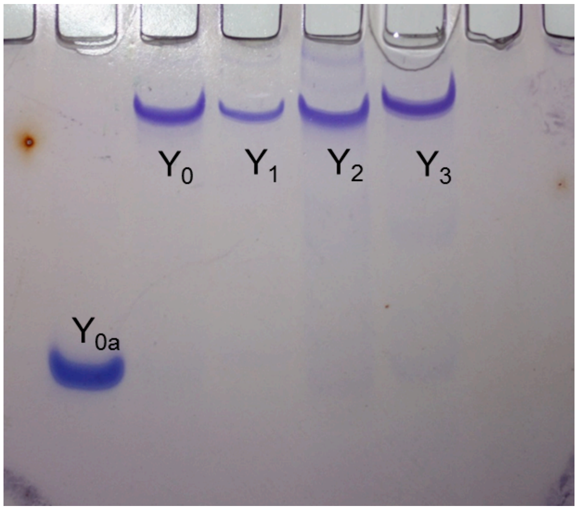

Figure S1. Native PAGE analysis of Y_0, Y_1, Y_2, $Y_{3.}$

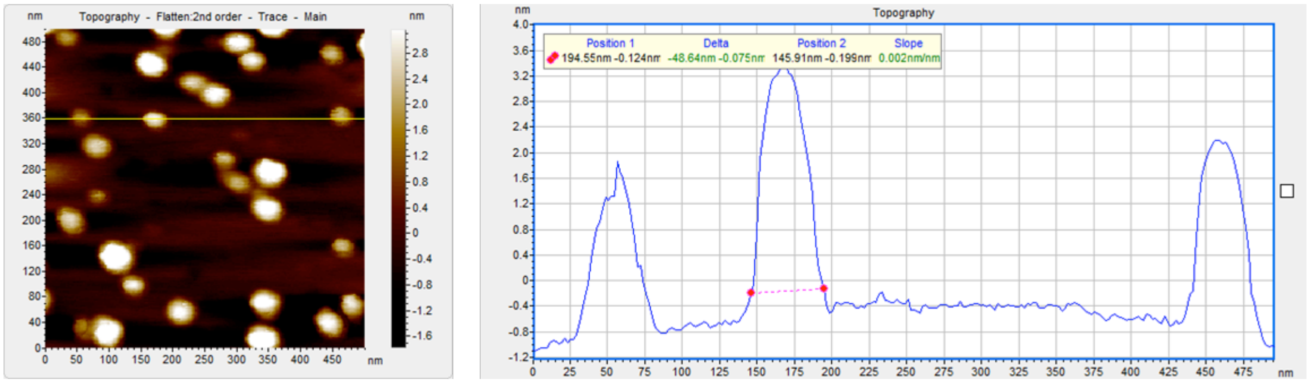

Figure S2. AFM imaging for the last generation ($G_{3\text{-}sgc8}$).

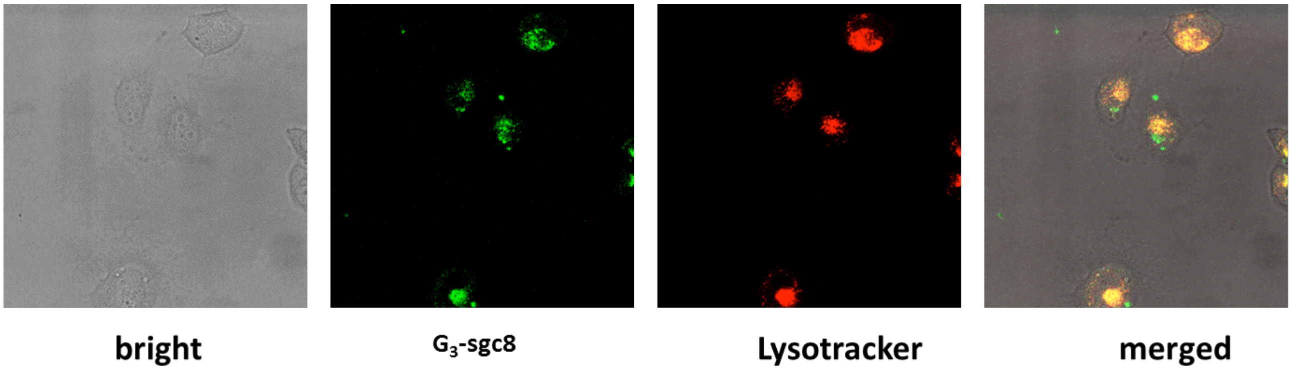

Figure S3. Microscopy images of the colocalization of FITC-$G_{3\text{-}sgc8}$ and Lysotracker (lysosome marker), indicating that the internalized $G_{3\text{-}sgc8}$ was localized in the lysosome.

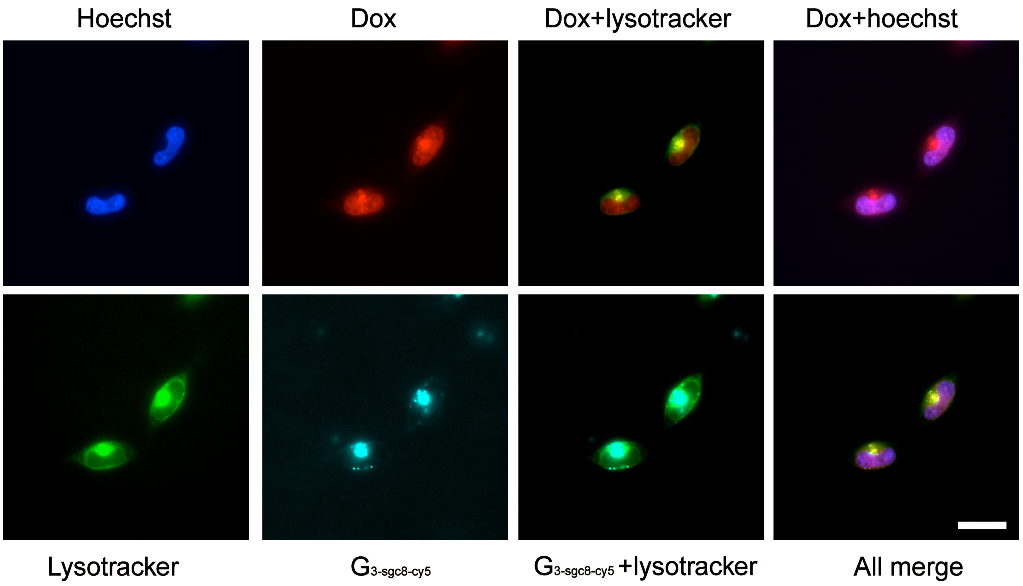

Figure S4. Subcellular distribution of Dox (red) loaded $G_{3\text{-sgc8-cy5}}$ (cyan). Hoechest (blue) and Lysotracker (green) were used to stain the cell nuclei and acidic organelles. Cells were imaged using a 60x oil-immersion objective. The merged pictures were used to indicate that drugs have started to diffuse into nuclei within 30min incubation. Scar bar: 20um.

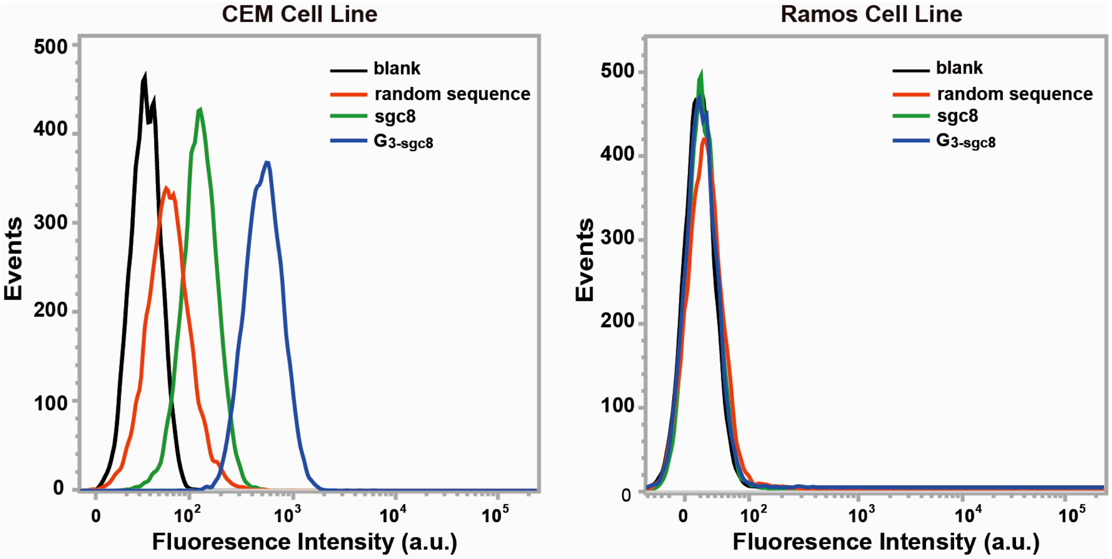

Figure S5. Flow cytometeric analysis after incubating $G_{3\text{-sgc8}}$ with CEM and Ramos cell lines for 2 hours, demonstrating that $G_{3\text{-sgc8}}$ had strong selective cell recognition.

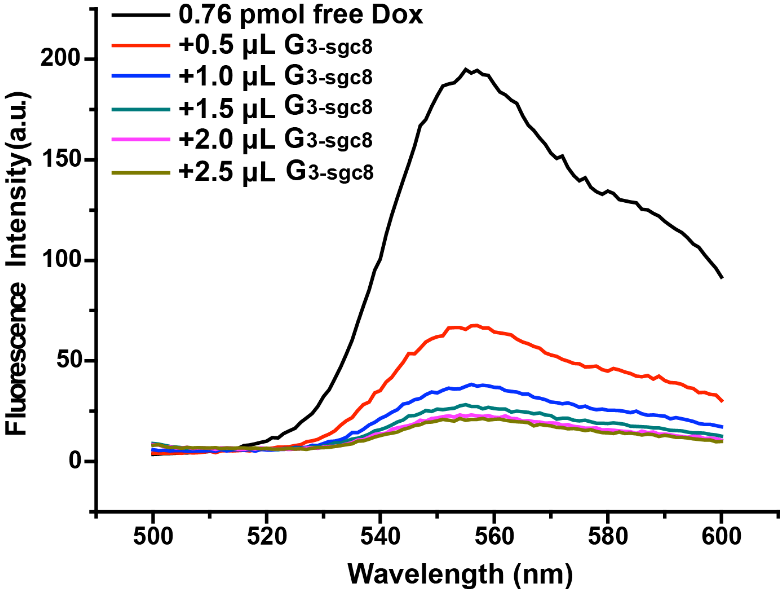

Figure S6. The fluorescence spectra of the Dox solution after adding different amounts of $G_{3\text{-sgc8}}$. Due to FRET between Dox and double-stranded DNA, the change of fluorescence intensity can be used to evaluate the max drug amount in $G_{3\text{-sgc8}}$.

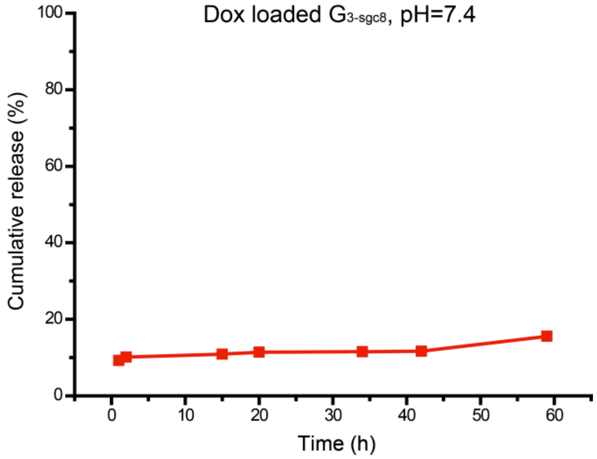

Figure S7. Cumulative release of Dox loaded in $G_{3\text{-sgc8}}$ in 60 hours.

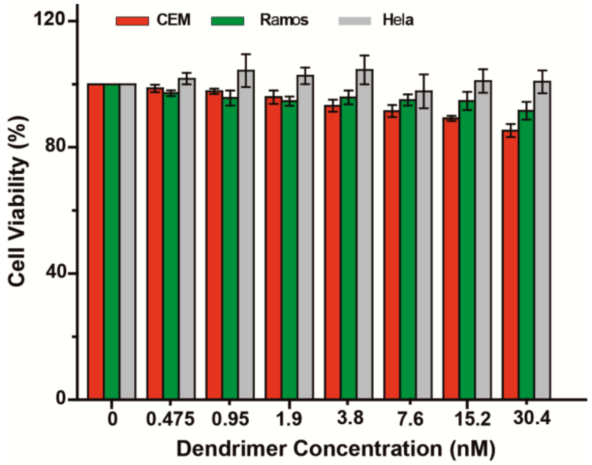

Figure S8. MTS assay results verifying the biocompatibility of $G_{3\text{-sgc8}}$ in CEM cells, HeLa cells and Ramos cells.
